# A Study on Efficacy of UGI Scopy in Cholelithiasis Patients before Laparoscopic Cholecystectomy

**DOI:** 10.1155/2021/8849032

**Published:** 2021-01-13

**Authors:** Supreeth Kumar Reddy Kunnuru, B. Kanmaniyan, Manuneethimaran Thiyagarajan, Balaji K. Singh, Nitesh Navrathan

**Affiliations:** General Surgery Department, Sri Ramachandra Medical University, Chennai, India

## Abstract

**Objectives:**

Upper abdominal symptoms are common in both gallstone disease and inflammatory disorders of gastroduodenum. To differentiate the causes of upper gastrointestinal symptoms due to gallstone and gastroduodenal disorders, upper gastrointestinal (UGI) scopy is a useful diagnostic tool. Our aim of study is to determine the efficacy of the preoperative UGI scopy and concurrent treatment of associated esophageal and gastric pathologies with symptomatic cholelithiasis in view of postoperative symptom reduction.

**Materials and Methods:**

This is a prospective study comprising 400 symptomatic cholelithiasis patients admitted in our institution. All patients underwent upper GI endoscopy (1–4 days) prior to cholecystectomy, and the findings were noted. Then, based on findings in UGI scopy, patients were grouped as group A (endoscopy normal) and group B (endoscopy with some findings). Group B patients were treated with medication, and both groups were operated with laparoscopic cholecystectomy. Pain and other symptoms in the preoperative period and postoperative period were measured and compared in both groups.

**Results:**

After excluding 7 patients with significant endoscopy findings, we have included 400 patients who underwent laparoscopy cholecystectomy. In a total of 400 patients, median age of presentation was 47.3 and female to male ratio was 2.2 : 1. Endoscopy showed some pathological findings in 75.5% patients, and the commonest endoscopy finding was gastritis. On comparison of pain score in preoperative patients, pain score was high in group B patients (*p* < 0.05). Pain reduction was significant in postoperative 1st, 4^th^, and 6th weeks in both groups (*p* < 0.0005). In the same way, other symptoms other than pain were compared which shows postoperative symptom reduction is highly significant in group B patients.

**Conclusion:**

Clinical presentation of cholelithiasis and other upper GI diseases resemble each other. It is difficult to discriminate between upper GI symptoms due to cholelithiasis or any other upper GI conditions. Although UGI scopy is not recommended for all patients with cholelithiasis, it may be beneficial to do UGI scopy in certain cholelithiasis patients with atypical presentation to prevent atypical symptoms after surgery.

## 1. Introduction

Upper abdominal symptoms are common in both gallstone disease and inflammatory disorders of gastroduodenum. Although there are a lot of geographic variations in gallstone disease, it is in high prevalence rate in developed countries [1, 2]. Gallstone disease is one of the common biliary pathologies, but majority of the gallstone diseases are asymptomatic.

High rate of ultrasound abdomen is another cause of increase in diagnostic rate of asymptomatic gallstone disease [3, 4]. Only 1-2% of asymptomatic gallstone patients develop symptoms every year and require laparoscopic cholesystectomy surgery.

To differentiate the causes of upper GI symptoms due to gallstone and gastroduodenal disorders, upper GI scopy is a useful diagnostic tool, but there are conflicting evidences to say UGI scopy as routine investigation for all cholecystectomy patients for treating medically treatable upper GI pathology [5]. The underlying correlation of symptoms of these two conditions is not well established yet [6].

The group of patients with cholelithiasis with nonspecific upper GI symptoms can present with the same type of symptoms even after cholecystectomy, and it is called as postcholecystectomy syndrome. This syndrome usually due to diseases which are unrelated to cholelithiasis-like gastritis, esophagitis, peptic ulcer disease, and hiatus hernia [7]. It is therefore important that an accurate documentation of atypical abdomen pain be made, and patients should be treated together with surgery for cholelithiasis. Cholecystectomy surgery in patients with gallstone and nonspecific symptoms is unjustifiable [8], so preoperative documentation of upper GI pathology is important before doing laparoscopic cholecystectomy.

Few studies show the reason for the postcholecystectomy persistent symptoms and importance of UGI scopy before surgery. There is no study to find out the effect of concurrent medical treatment for oesophagogastric pathology before doing cholecystectomy in postoperative symptoms reduction.

### 1.1. Aim and Objectives

1. To evaluate the value of UGI scopy as a routine investigative tool prior to cholecystectomy in symptomatic cholelithiasis patients
2. To determine the advantages of the preoperative diagnosis and concurrent treatment of associated esophageal and gastric pathologies with symptomatic cholelithiasis in view of postoperative pain and other symptoms reduction

## 2. Materials and Methods

In this prospective study, 407 patients with symptomatic cholelithiasis admitted in Sri Ramachandra Medical Center, Chennai, between February 2017 and October 2018 were initially included.

Age below 18 years, associated complications like pancreatitis, choledocholithiasis, cholangitis, and cholecystitis were excluded. All patients with proven gastroduodenal esophagus disorders with previous UGI scopy were excluded from study. Ultrasound findings like acute or chronic cholecystitis, empyema gallbladder, and gallstone pancreatitis were excluded. Ultrasonography findings other than cholelithiasis like gallbladder polyps, adenomyomatous, and carcinoma of gallbladder were excluded, and postoperative complications like CBD injury and bilioma were also excluded.

All patients admitted with cholelithiasis underwent upper GI endoscopy (1–4 days) prior to cholecystectomy, and the findings were noted. Based on UGI scopy, patients were divided into two groups:

  In group A: symptomatic cholelithiasis patients with UGI scopy normal were included
  In group B: symptomatic cholelithiasis patients with UGI scopy show some pathological findings were included

In group B patients, according to the UGI scopy findings, medical management was started as per American college of gastroenterology guideline:

1. We have started PPI and mucosal coating agents for gastritis, gastric erosion, and gastric ulcer diseases 3-4 days before surgery and continued totally 6 weeks.
2. We gave PPI with domperidone/lesuride (prokinetic drugs) for reflux esophagitis and lax lower end of esophagus patients 3-4 days before surgery and continued for 6 weeks.
3. *H. pylori*-positive patients were started with *H. pylori* kits for 2 weeks followed by PPI for 6 weeks.

7 patients in group B had severe gastroduodenal symptoms and not improved with medical treatment. Treating physician decided these 7 patients would need long-term medical treatment before surgery and so were excluded from the study. Rest of 400 patients were only included in the study and proceeded laparoscopic/open cholecystectomy in all patients.

We considered all 400 patients as symptomatic cholelithiasis although group B alone shows findings in UGI scopy. So after starting medical treatment in group B, both groups of patients were operated with laparoscopic cholecystectomy and postoperative follow-up was done for 6 weeks. Patient's pain score was assessed in both groups preoperatively, and comparison was done between both groups. Pain score was assessed with pain analogue scale 0–5.

Postoperative pain score was assessed in the 2nd week, 4th week, and 6th week in both groups. The comparison of pain score was done between these two groups.

Symptoms other than pain like heart burns, nausea, vomiting, dyspepsia, dysphagia, and weight loss were also collected together in both groups in the preoperative period and 6th week of postoperative period, and comparison was made.

### 2.1. Software Analysis

The collected data were analyzed with IBM SPSS statistics software 23.0 Version. To describe about the data descriptive statistics, mean, median, IQR, and S.D. were used.

To find the significant difference between the bivariate samples in independent groups (group A and group B), the Mann–Whitney *U* test was used, and for the repeated measures (Pre, 1st, 4^th^, and 6th week), the Friedman test followed by the Wilcoxon signed rank test was used. In all the above statistical tools, the probability value 0.05 is considered as the significant level.

### 2.2. Observation and Results

Since all included patients were symptomatic, UGI scopy was done for all 400 patients. In 302 (75.5%) patients, some pathologies were noted and this group of patients was included in group B. Only 98 (24.5%) patients who underwent UGI scopy were completely normal, and this group of patients was included in group A.

Age distribution of cholelithiasis is compared in Table 1. The mean age was found to be 45.3 years with a standard deviation of +22.29 years. Maximum number of patients in group B was in 51–60 years of age group (26.4%). In 400 patients, there were 276 (69%) female patients and 124 (31%) male patients.

All symptoms were evaluated in all preoperative patients and tabulated.

According to Table 2, pain abdomen is the commonest symptom (99% of patients) followed by heart burn (25.5%) and dyspepsia (23%). Since UGI scopy was normal, we considered symptoms for all patients in group A were due to gallstone only, but group B symptoms were considered due to combinations of gallstone disease and gastrointestinal problems.

Table 3 shows UGI scopy findings in group B patients. In some patients, more than one finding was noted. Maximum finding noted was gastritis (22%), and the next common finding was gastric erosion (19%).

Table 4 shows the pain score (0–5) in preoperative patients and 1st week, 4th week, and 6th week of postoperative patients in each group. No patient presented with a pain score of 5.

Table 5 shows bivariate comparison between groups A and B. It shows no statistical significant difference in the 4th week pain score (*p* value of 0.453) and 6th week pain score (*p* value of 0.306), whereas the comparison of pre-op pain score (*p* < 0.05) shows statistical significance and 1st week pain score (*p* < 0.0005) shows statistically high significant value.

In Table 6, the multivariate analysis by the Friedman test in the group A and group B individually between the preoperative period and postoperative weeks shows highly statistical significance of pain reduction with *p* < 0.0005.

Bivariate analysis within the groups also showed highly statistical significance with *p* < 0.0005 in pre-op and post-op, all possible weeks of within group A and group B.

**Figure 1:**
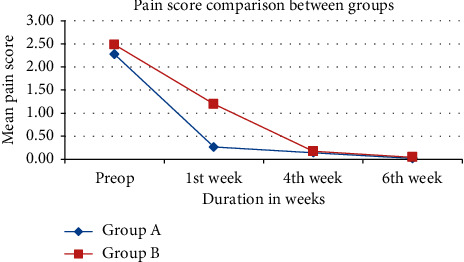
Comparison of pain score between two groups.

Figure 1 shows the comparison of pain reduction from the preoperative period to the 6th week of postoperative period in both groups. In the 4th and 6th weeks, pain reduction in both groups is equal.

In Table 7, comparing each symptom with preoperative patients of both groups shows significantly all symptoms are high in group B patients. Comparing each preoperative symptom with postoperative 6th week symptom (within the group), significant symptoms reduction is seen in both groups (group A, *p* < 0.05; group B, *p* < 0.0005).

## 3. Discussion

In our study, totally 400 patients presented with symptomatic cholelithiasis. Mean age of presentation was 45.3%, while in Kim et al.'s study, the mean age of presentation was 47.3 ± 10.9 years [9].

The female to male ratio of our patients was 2.2 : 1. In Kim et al.'s study, the female to male ratio was 1.4 : 1 [9]. Another study conducted for gender ratio of cholelithiasis in Novacek showed 2-3 times higher incidence rate in female because of estrogen hormonal effect [10].

According to Fitzgerald et al., upper abdominal pain, dyspepsia, and nausea and vomiting are the common symptoms of gallstone disease [11]. In our study, pain abdomen was the commonest symptom (99% of patients) followed by heart burn (25.5%) and dyspepsia (23%).

In our study, we got upper GI scopy positive findings in 75.5% of our total patients. Only 24.5% of patients presented with normal UGI scopy. It means 3/4th of cholelithiasis patients were associated with other gastroduodenal problems. This is the key point in our study. If the treatment was not started for this finding, patients in group B will have persistent pain even after cholecystectomy. On the contrary, 1/4th of the patient underwent UGI scopy in normal findings, so we cannot absolutely recommend the endoscopy for all symptomatic cholelithiasis patients.

In Ayuo et al.'s study [12], common findings in upper GI scopy were gastric ulcer (3.1%), duodenal ulcer (11%), gastritis (8.4%), duodenitis (5%), and reflux esophagitis (7.9%).

In our study, gastritis (22%), gastric erosion (19%), reflux esophagitis (12%), lax lower end of esophagus (10%), and gastric and duodenal ulcer (7%) were comparable to the above study.

Since pain was the major symptom in both groups, statistical analysis was done between the pain score of the pre-op patients in both groups A and B separately (Table 4). The pre-op pain score was comparatively high in group B (*p* value <0.05). In group B, 22.5% of patients presented with a pain score of 4, but it was 0% in group A. In addition to this, 27% of group B and 5% of group A presented with a pain score of 3. It means pain was comparatively high in UGI finding patients than UGI scopy normal patients.

In the 1st week (Table 4), the pain score was significantly high in group B patients (*p* < 0.0005). In group B, pain score 3 was seen in 20.5% of patients and pain score 4 was seen in 1.5% of patients, but in group B, it was 0%. In the 4th week and 6th week, there was no difference in pain score in between groups A and B (*p*=0.453,  *p*=0.306). It means because of medical management in the 4th week and 6th week, pain reduction in group B was equal to group A. It means group B patients were treated according to their UGI scopy findings for 4 to 6 weeks helping in maximum pain reduction which is comparable to group A-UGI scopy normal patients.

In addition to these multivariate and bivariate analyses of pre-op and post-op periods of 1st, 4^th^, and 6th weeks, pain score in group A as well as group B shows significant pain reduction in each week. *p* value for all analysis is less than **0.0005**. It means that, in both groups, gradual pain reduction is significant in all weeks.

Figure 1 clearly explains pain reduction rate in both groups equal in the 4th week and 6th week although there was a delay in the 1st week for group B.

In our study, at the end of 6 weeks, 96% of the total postoperative patients became completely pain free. Similar study done by Khedkar et al. [12] showed that pain subsided completely by the end of 3 months. The overall response rate was 95% at the end of 3 months.

While comparing other symptoms excluding pain, there was significant reduction in both groups of patients after surgery, but in group B, it was highly significant (*p* < 0.0005). It showed the effect of concurrent medical treatment in group B is effective in controlling postcholecystectomy symptoms (excluding pain) also.

Rashid [14] shows benefit of UGI scopy for patients undergoing laparoscopic cholecystectomy. In his study, one group of patients underwent UGI scopy before surgery and treatment started according to the endoscopy findings. For the other group of patients, endoscopy was not done. The result showed persistence of symptoms in 32.7% patients who were not scoped and only 3.3% had persistence of pain in patients scoped and treated. Similarly, in our study, for all scoped and treated patients pain resolved almost equal to patients with normal endoscopy.

Rassek et al. [15] recommend UGI scopy before elective cholecystectomy, and in their study, 11.3% of patients underwent a change in plan of therapy because of findings in UGI scopy. In our study, actually, we have excluded 7 patients with significant problems in endoscopy findings. We have recommended only medical treatment. In Schwenk et al. [16] 93.1% of patients underwent UGI scopy before cholecystectomy and 30.2% of patients had pathological findings. In addition to this, 2.5% of patients underwent additional GI surgical procedure along with cholecystectomy based on findings. In our study, in 75.5% patients, we found some findings in endoscopy, but we did not proceed any additional surgical procedure along with cholecystectomy.

Diettrich et al. [17] show 31% of patients had abnormal OGD findings resulting in change in plan in therapy. Thybusch et al. [18] show therapeutic implications of routine OGD before cholecystectomy. In their study, 8.3% of patients' OGD findings influenced management and surgery was postponed awaiting medical management. Two patients underwent gastrectomy for gastric cancer. In our study, although preoperative endoscopy did not change the plan of treatment, it helped for concurrent treatment of other UGI diseases. There is no malignancy detected in our UGI scopy.

Sosada et al. [19] recommended pan-endoscopy for all cholecystecomy patients, surgery was delayed for ulcer patients, and they were treated appropriately before surgery. In their study, 16 patients completely became asymptomatic after medical treatment and cholecystectomy was not performed. In our study, we did not include the 7 patients who had severe gastric ulcer. We recommend only medical treatment.

In contrast, Lemberts et al.'s meta-analysis [20] concluded that, despite high diagnostic value for UGI scopy, its value as a tool to prevent gallbladder surgery is limited. This meta-analysis was done with 12 cohort studies. The estimated abnormality detected in UGI scopy was 36.3%, and only 3.8% of patient's surgery was avoided, but they concluded that UGI can be done only for the cholelithiasis patient undergoing surgery. In our study, since we planned for surgery in all patients, we did UGI scopy in all patients.

In this study, for all patients in group B, medical management was started together and surgery was proceeded. The usual comment is why surgery was done for all patients in group B instead of giving medical treatment alone since UGI scopy shows some findings? Actually, in group A, all patients (98) are proved as symptoms due to gallstones alone, but in group B (302), we cannot prove the symptoms due to UGI pathology alone or due to cholelithiasis alone. So we considered group B patients' symptoms due to variable combination of both diseases. Although UGI scopy showed finding in group B patients, we could not predict cholelithiasis as asymptomatic in all group B patients. In addition to this, we excluded the 7 patients who had predominant UGI problems who needed long-term medical treatment and were not suitable to proceed surgery. We included only 400 patients who were going for surgery for cholelithiasis.

The obstacles with routine UGI scopy for all patients are cost of the procedure, waiting list, patient discomfort, and complications due to endoscopy. However, the advantage of this study is by doing routine UGI scopy, and we can rule out other upper GI diseases including malignancy for all patients. It can also prevent emergency UGI scopy for group B patients which is more expensive because it needs hospital admission.

In addition, our study excluded cholecystitis, empyema gallbladder, and gallstone pancreatitis patient. So patients with confirmed gallbladder pathology with gallstone were directly proceeded with surgery instead of doing UGI endoscopy before surgery.

One-fourth of patient in our study showed normal endoscopy, so we cannot also completely recommend UGI scopy for all cholelithiasis patients with symptoms. However, we may recommend UGI scopy for patients with atypical presentation to rule out other causes of pain to prevent persistent symptoms even after surgery. It is also important to evaluate thoroughly all cholelithiasis patients' preoperative period to prevent cholecystectomy in asymptomatic cholelithiasis patients as prophylactic cholecystectomy is not an acceptable procedure.

We want to clarify that we did not operate any silent cholelithiasis patient. Main inclusion criteria in our study are symptomatic cholelithiasis. This means pain in the right hypochondriac region or referred pain to right shoulder and colicky pain. Our main aim of the study is to say even in confirmed symptomatic cholelithiasis patients, there may be some associated UGI pathologies like gastritis and reflex esophagitis which needs simultaneous treatment to prevent postsurgery symptoms. The other is to explain both UGI pathology and gallbladder pathology can coexist and one may be predominant to present symptoms, but we have to treat both if present together (our group B patients fit into this category).

## 4. Conclusion

Clinical presentation of cholelithiasis and other upper GI diseases resemble each other. It is difficult to discriminate between upper GI symptoms due to cholelithiasis or any other upper GI conditions. In many patients with cholelithiasis, upper GI symptoms are not completely alleviated even after surgery which may need further investigations.

Although UGI scopy is not recommended for all patients with cholelithiasis, it is beneficial to do UGI scopy in certain cholelithiasis patients with atypical presentation to prevent atypical symptoms after surgery.

## Figures and Tables

**Table 1:** Comparison of age and sex distribution of symptomatic cholelithiasis and comparison of group A and B patients number.

Age	Total no. of cases	Group A % within group	Group B % within group
18–30	96 (24%)	26 (26.5%)	70 (23%)
31–40	80 (20%)	20 (20.4%)	60 (19.8%)
41–50	60 (15%)	18 (18.3%)	42 (14%)
51–60	100 (25%)	20 (20.4%)	80 (26.4%)
61–70	40 (10%)	8 (8%)	32 (10.5%)
71–80	24 (6%)	6 (6%)	18 (6%)
Total	400 (100%)	98 (24.5%)	302 (75.5%)
Male	124 (31%)	28 (28.5%)	96 (31.7%)
Female	276 (69%)	70 (71.4%)	206 (68.2%)

**Table 2:** Preoperative symptoms in all symptomatic cholelithiasis patients.

Pain abdomen	Heart burn	Nausea and vomiting	Dyspepsia	Chest pain	Dysphagia
387 Group A = 86 Group B = 301	102 Group A = 21 Group B = 81	34 Group A = 12 Group B = 22	92 Group A = 28 Group B = 64	3 Group A = 0 Group B = 3	11 Group A = 2 Group B = 9
99%	25.5%	8.5%	23%	0.7%	2.7%

**Table 3:** Comparison of UGI scope findings in group B patients.

Gastritis	Gastric erosion	*H. pylori* positive	Gastric and duodenal ulcer	Esophagitis	Lax LE
90	76	54	30	49	40
22%	19%	13%	7%	12%	10%

**Table 4:** Comparison of preoperative and postoperative pain score in group A and group B patients (% within group).

Pain score	Preoperative pain score		1st week of post-op day		4th week of post-op day		6th week of post-op day	
	Group A (*n* = 98) patient	Group B (*n* = 302) patient	Group A (*n* = 98) patient	Group B (*n* = 302) patient	Group A (*n* = 98) patient	Group B (*n* = 302) patient	Group A (*n* = 98) patient	Group B (*n* = 302) patient
0	5 (5%)	8 (2%)	72 (73.4%)	45 (14.9%)	84 (85.7%)	256 (84.7%)	96 (97.9%)	289 (95.6%)
1	13 (13.2%)	20 (66.2%)	26 (26.5%)	167 (55.2%)	14 (14.2%)	46 (15.2%)	2 (2%)	13 (43%)
2	47 (48%)	142 (47%)	0 (0%)	82 (27.1%)	0 (0%)	0 (0%)	0 (0%)	0 (0%)
3	33 (33.6%)	98 (35%)	0 (0%)	6 (1.9%)	0 (0%)	0 (0%)	0 (0%)	0 (0%)
4	0 (0%)	34 (11.2%)	0 (0%)	0 (0%)	0 (0%)	0 (0%)	0 (0%)	0 (0%)

**Table 5:** Comparative analysis of pain score in group A and group B patients in pre-op and postoperative period.

Bivariate comparison of groups by Mann–Whitney *U* test								
Variables	Pre-op		1st week		4th week		6th week	
	Group A	Group B	Group A	Group B	Group A	Group B	Group A	Group B
Mean	2.28	2.48	0.27	1.20	0.14	0.18	0.02	0.04
Median	2.00	2.00	0.00	1.00	0.00	0.00	0.00	0.00
SD	0.670	0.776	0.444	0.705	0.352	0.381	0.142	0.203
IQR	1	1	1	1	0	0	0	0
*Z* value	1.937		10.755		0.751		1.024	
*p* value	0.05^*∗*^		0.0005^*∗∗*^		0.453^#^		0.306^#^	

^*∗∗*^Highly significance at *p* < 0.01; ^#^no significance at *p* > 0.05.

**Table 6:** Multivariate analysis in group A and group B.

Multivariate comparison of weeks in groups by Friedman test						
Mean		Median	SD	IQR	Friedman *x*^2^	*p* value
*Group A*						
Pre-op	2.28	2.00	0.670	1	263.036	0.0005^*∗∗*^
1st week	0.27	0.00	0.444	1		
4th week	0.14	0.00	0.352	0		
6th week	0.02	0.00	0.142	0		

*Group B*						
Pre-op	2.48	2.00	0.776	1	852.937	0.0005^*∗∗*^
1st week	1.20	1.00	0.705	1		
4th week	0.18	0.00	0.381	0		
6th week	0.04	0.00	0.203	0		

^*∗∗*^Highly statistical significance at *p* < 0.01.

**Table 7:** Comparison of other symptoms excluding abdominal pain.

	Group A pre-op	Group B pre-op	Group A 6th week post-op	Group B 6th week post-op
Heart burn, *N* = 96	16 (16.6%)	80 (83.3%)	3 (3%)	5 (5.2%)
Nausea and vomiting, *N* = 6	1 (16%)	5 (83%)	0 (0%)	0 (0%)
Dyspepsia, *N* = 92	6 (6.5%)	86 (93.4%)	1 (1%)	6 (6.5%)
Chest pain, *N* = 3	0 (0%)	3 (100%)	0 (0%)	0 (0%)
Dysphagia, *N* = 11	1 (9%)	10 (90%)	0 (0%)	1 (9%)

## Data Availability

All data are available in hospital records, and access is restricted for patients' privacy and ethical concern.

## Abbreviations

GI: Gastrointestinal
UGI: Upper gastrointestinal
PPI: Proton pump inhibitor
OGD: Oesophago-gastroduodenoscopy
H. pylori: *Helicobacter pylori*.
